# Asymmetric Drug-Induced Parkinsonism and Psychopathology: A Prospective Naturalistic Study in Long-Stay Psychiatric Patients

**DOI:** 10.3389/fpsyt.2018.00018

**Published:** 2018-02-05

**Authors:** Lydia E. Pieters, P. Roberto Bakker, Peter N. van Harten

**Affiliations:** ^1^Faculty of Medicine, University Medical Center Utrecht, Utrecht University, Utrecht, Netherlands; ^2^Psychiatric Center GGz Centraal, Amersfoort, Netherlands; ^3^Department of Psychiatry and Psychology, Maastricht University Medical Center, South Limburg Mental Health and Teaching Network, Maastricht, Netherlands

**Keywords:** parkinsonism, movement disorders, asymmetry, psychopathology, schizophrenia, psychotic disorders

## Abstract

**Background:**

Drug-induced parkinsonism (DIP) is the most common movement disorder induced by antipsychotics. Although DIP is mostly symmetric, asymmetric DIP is reported in a substantial part of the patients. We investigated the frequency of motor asymmetry in DIP and its relationship to the severity of psychopathology in long-stay psychiatric patients.

**Methods:**

We obtained data from a cohort study of 207 long-stay psychiatric patients on the frequency and risk factors of tardive dyskinesia, akathisia, tardive dystonia, and DIP. From July 2003 to May 2007 (mean follow-up, 1.1 year) drug-induced movement disorders were assessed at least two times in each patient, with a frequency of persistent DIP of 56.2%. All patients who had at least one time parkinsonism in the upper/lower limb(s) were included for analyses (190 patients, 79 women; mean age, 48.0 ± 12.9 years). The Unified Parkinson Disease Rating Scale motor scale was used to calculate the frequency of asymmetric parkinsonism. Multilevel mixed models were built to explore the relationship between asymmetry in parkinsonism and the severity of psychopathology, measured on the Clinical Global Impression-Schizophrenia scale severity index (CGI-SCH SI).

**Results:**

The frequency of asymmetric parkinsonism was 20.8%. Asymmetry in parkinsonism was associated with symptom severity on all CGI-SCH SI scales (β range, 0.37–3.74) and significantly associated with the positive symptom scale (β, 3.74; 95% CI, 0.35–7.31).

**Conclusion:**

DIP is asymmetric in a substantial part of patients. Asymmetric presentation of DIP is of clinical relevance as it is related to the severity of psychopathology and may alert the clinician of more severe psychopathology. Future research is recommended to provide insight into the neuropsychopathology and clinical value of asymmetric parkinsonism for psychiatric patients.

## Introduction

Parkinsonism is the most frequently observed movement disorder induced by antipsychotics (1). In chronic psychiatric patient populations, reported prevalence rates of drug-induced parkinsonism (DIP) range from 17 to 72% (2–4). The estimates for the prevalence and incidence of DIP vary widely, reflecting differences in study populations and methodology, such as criteria for diagnosis and length of observation. Furthermore, true prevalence rates may be even higher than reported in these studies, as the disorder is frequently unrecognized or misdiagnosed as idiopathic Parkinson’s disease (iPD) (5–7). DIP is particularly burdensome in psychiatric patients, as it can result in significant morbidity, disability, and treatment non-compliance (6, 8, 9). Therefore, recognition of the clinical presentation of DIP in psychiatric patients is essential for adequate diagnosis and treatment (9).

Drug-induced parkinsonism is defined as an akinetic-rigid syndrome, most commonly induced by the use of antipsychotic agents (5, 10). All types of antipsychotics, including second-generation antipsychotics (SGAs), have the potential to induce parkinsonism (11). High affinity for the dopamine-2 (D2) receptor and high dosage of the antipsychotic are associated with the risk for development of parkinsonism (12–17). Older age is the most obvious risk factor for DIP, possibly reflecting the age-related decline in number of D2 receptors in the striatum, although some studies report a higher risk in younger patients (10, 18–25). Other individual risk factors for DIP include the female gender (21, 26–28), cognitive impairment (29, 30), and possibly a genetic predisposition (31–34). Patients usually develop DIP several days to weeks after starting an antipsychotic or increasing the dose, but it can take several months or more before symptoms appear (5, 26). Although the clinical presentation of DIP is classically described as symmetric parkinsonism, usually without tremor at rest, 30–50% of the patients with DIP present with unilateral parkinsonism and/or rest tremor (5, 10). Therefore, the clinical features of DIP overlap with iPD, characterized by unilateral symptom onset and persisting asymmetry of symptoms throughout disease progression (35). However, as slowly and progressively worsening of symptoms is a clinical hallmark of iPD, DIP is expected to remain relatively stable over time, if treatment of the offending drug remains unchanged (35–37). After cessation of the drug, DIP resolves in the majority of patients within a few weeks to months (28, 38). Nevertheless, in some patients, symptoms persist, or even worsen, after drug withdrawal, suggesting the development of concomitant iPD (39, 40). It has been suggested that DIP may be less reversible after cessation of the antipsychotics, as thought before, owing to irreversible neurotoxic effects of long-term exposure to antipsychotics. This hypothesis was supported by preliminary findings in animal studies (41, 42).

Considering the above-mentioned differences in symptom presentation of DIP, it may be questioned if the pathomechanism of DIP is the same for all patients, especially in case of asymmetric parkinsonism. DIP is expected to appear symmetrically because the offending drug is acting equally on both sides of the brain. Therefore, patients with asymmetric DIP might have inherent neurochemical asymmetry of the brain. Several studies have addressed the occurrence of asymmetric DIP although its precise pathomechanism is not understood (6, 43, 44). Some cases of asymmetric DIP may be explained by preclinical iPD, unmasked by the use of antidopaminergic drugs (39, 40). However, given the large gap between the incidence of iPD and DIP in patients using antipsychotic agents, it is very unlikely that all cases of asymmetric DIP can be explained by this theory (2, 26, 35). Furthermore, normal findings of single-photon emission computed tomography scans with dopamine transporter ligands in patients with asymmetric DIP support the absence of iPD in these patients (45, 46). It has been suggested that other factors than asymmetric loss of dopaminergic neurons could account for the asymmetric symptom presentation, such as postsynaptic imbalance of dopamine receptors (45). Another explanation is pre-existing unequal functional reactivity of the basal ganglia (43).

If asymmetric DIP indeed reflects neurochemical asymmetry in the brain, asymmetric DIP could be of clinical relevance, as asymmetric structure and functioning in the brain has been associated with the severity of psychopathology in several neuropsychiatric disorders, including affective and psychotic disorders (47–52). It could be suggested that not only brain asymmetry but also asymmetric motor symptoms can be related to the severity of psychopathology in psychiatric patients. Therefore, we hypothesize that asymmetry of motor symptoms in DIP is associated with the severity of psychopathology in psychiatric patients. To our knowledge, the association between asymmetric parkinsonism and psychopathology has not been investigated before. In the current cohort of long-stay patients, the aim of this study is to (i) estimate the frequency of motor asymmetry in DIP and (ii) examine whether asymmetry in parkinsonism is related to the severity of psychopathology.

## Materials and Methods

### Subjects

Data originated from a cohort of 207 patients (mean age, 47.4 ± 12.8 years, 87 women) with chronic mental illness from a general psychiatric hospital (GGZ Centraal, Amersfoort, the Netherlands). The protocol was approved by the medical ethical committee, and written informed consent was obtained from each patient. Over the course of 4 years (July 2003–May 2007), with a mean (SD) period of observation of 1.1 (0.64), patients had at least two assessments of drug-induced movement disorders, namely tardive dyskinesia, akathisia, tardive dystonia, and parkinsonism. From this cohort, we included all patients with the presence of parkinsonism in the upper and/or lower limb(s) in at least one measurement. Full details of the study design and assessment of movement disorders can be found in a previous publication (18).

### Measures

Movement disorders (tardive dyskinesia, akathisia, tardive dystonia, and parkinsonism) were assessed at baseline and each follow-up assessment by a trained psychiatrist, using a standard protocol (4). Parkinsonism was assessed on the Unified Parkinson Disease Rating Scale (UPDRS), motor scale (14 items, scored from 0 to 4) (53). Mean values of the right and left scores of the upper and lower limbs (UPDRS items, 20–26: 20–21 tremor, 22 rigidity, 23–26 bradykinesia) were calculated, and then the symmetry index was calculated according to the following formula: symmetry index = |right − left UPDRS score|/(right + left UPDRS score) (54, 55). The symmetry index ranged from 0 to 1, with higher values indicating a higher degree of asymmetry. A cut-off value of ≥0.20 was used to estimate the frequency of asymmetric parkinsonism (56). Severity of psychopathology was evaluated at baseline and each follow-up assessment using the Clinical Global Impression-Schizophrenia scale severity index (CGI-SCH SI), containing five subscales (positive, negative, depressive, cognitive, and global, scored from 1 to 7) (57).

Other variables extracted for the current study were age, sex, diagnosis according to DSM-IV, ethnic group (white or non-white), and duration of hospitalization, collected at baseline from patients’ case notes. At baseline and each follow-up assessment, the use of antipsychotic and anticholinergic medication at the time was collected from the hospital and outpatient pharmacy databases. As the data originated from a study that was conducted before the introduction of DSM-5, DSM-IV codes were used. The diagnosis “schizophrenia” hereafter refers to the DSM-IV codes 295.30, 295.10, 295.20, 295.90, 295.60, 295.70, and other diagnoses of “psychotic disorder” to 295.40, 297.1, 298.8, and 298.9.

### Statistical Analyses

We employed multivariate regression analyses for the associations between asymmetry in parkinsonism (symmetry index) and severity of psychopathology (CGI-SCH SI scales) for all measurements of the included patients (with parkinsonism in the upper/lower limb(s) in at least one measurement). Given the multilevel structure with repeated assessments clustered within the subjects, multilevel mixed models were built, using the MIXED routine of the Stata statistical program, with maximum likelihood estimation (58, 59). A random coefficient was included to allow the effect of the symmetry index to vary between subjects. Guided by previous literature, the following covariates were included in the model: age, sex, and diagnosis (schizophrenia or other) as time-independent variables, and dosage of antipsychotics (the defined daily dose), concomitant anticholinergic use (yes or no), and the presence of movement disorders (continuous measures of parkinsonism, dyskinesia, dystonia, and akathisia) as time-dependent variables. An interaction term between the covariates age and asymmetry in parkinsonism was introduced in the model. A quadratic term of the symmetry index was added to allow for a possible non-linear relationship between the symmetry index and severity of psychopathology.

The measures on the CGI-SCH SI scales were checked for normal distribution and allowed to be moderately skewed (60). Independent variables, including the symmetry index, could take almost any distribution, as the multilevel model with maximum likelihood estimation can handle these data well (60). Correction for multiple testing was not applied as this analysis concerned a hypothesis-driven approach, in which reduction of type I errors, with increase of type II errors and reduction of power as a consequence, was not of our main interest (61–63).

## Results

### Sample Characteristics

Table 1 shows the characteristics of the selected patients from the cohort, who had parkinsonism in the upper and/or lower limb(s) in at least one measurement (*N* = 190, women *N* = 79). Of the 190 patients, 181 (95.2%) and 113 (59.5%) patients completed the first and second follow-up assessment, respectively. All patients suffered from a chronic mental illness requiring long-term admission and had a history of cumulative antipsychotic intake of minimally 1 year. At baseline, the mean (SD) age of patients was 48.0 (12.9) years (range, 21.3–85.7 years), with a mean age at first admission of 25.0 (8.6) years. According to the DSM-IV criteria, 70.5% (*N* = 134) of the patients had a primary diagnosis of schizophrenia. Parkinsonism was the most prevalent persisting drug-induced movement disorder, with a persistence rate of 59.1% (*N* = 107). Over all measurements at baseline, first and second follow-up (number of measurements *N* = 477), the first-generation antipsychotic (FGA) and SGA use were 66.9 and 61.2%, respectively. Anticholinergics were concomitantly prescribed in 44.0% of all measurements (Table 2).

**Table 1:** Characteristics of patients with parkinsonism in the upper and/or lower limb(s)^a^ in at least one measurement (patients *N* = 190, number of measurements *N* = 484).

Variable	*N*	Mean (SD)	%
**Measurements at baseline**			
Age (years)	190	48.0 (12.9)	
Sex			
Female	190		41.6
Ethnicity			
Caucasian	190		85.8
DSM-IV classification			
Schizophrenia	190		70.5
Psychotic disorder			5.3
Affective disorder			13.7
Other/no diagnosis			10.5
Duration of admission (years)	190	22.7 (13.3)	

**Measurements at baseline and follow-up**			
CGI-SCH SI (range, 1–7)			
Positive	190^b^	2.7 (1.5)	
Negative		2.5 (1.4)	
Depressive		2.0 (1.1)	
Cognitive		2.3 (1.5)	
Global		2.8 (1.2)	
Persistence of movement disorders^c^			
Parkinsonism	181		59.1
Dyskinesia			28.7
Akathisia			5.0
Dystonia			5.0

*CGI-SCH SI, Clinical Global Impression-Schizophrenia scale severity index; UPDRS, Unified Parkinson Disease Rating Scale*.

*^a^The total of the items 20–26 of the UPDRS motor score is >0*.

*^b^Mean scores over the measurements at baseline and first and second follow-up of the 190 patients (number of measurements, *n* = 477) are displayed*.

*^c^Persistence of movement disorder: two consecutive positive assessments with a minimum interval of 3 months*.

**Table 2:** Antipsychotic use at the measurements at baseline and first and second follow-up (patients *N* = 190, number of measurements *N* = 477).

Variable	Mean (SD) or %
Dose (DDD)	2.5 (2.5)
Number of antipsychotics	
0	5.0
1	46.1
2	38.8
3	9.0
4	1.1
Generation	
FGA only	33.8
SGA only	28.1
Both	33.1
Concomitant anticholinergic use	44.0

*DDD, defined daily dose; FGA, first-generation antipsychotic; SGA, second-generation antipsychotic*.

### Frequency of Asymmetry in Parkinsonism

Symmetry of parkinsonism was evaluated for all measurements of parkinsonism in the upper and/or lower limb(s) (patients *N* = 190, number of measurements *N* = 404), as shown in Table 3. The mean (SD) symmetry index was 0.13 (0.24), which was the highest for the bradykinesia items: 0.16 (0.29). Asymmetric parkinsonism (symmetry index, ≥0.20) was present in 84 (20.8%) of the measurements.

**Table 3:** Symmetry of UPDRS motor scores in the measurements with parkinsonism in the upper and/or lower limb(s)^a^ (patients *N* = 190, number of measurements *N* = 404).

UPDRS motor symmetry, items 20–26	Mean (SD)	Range	Number of measurements
Score per UPDRS item	0.64 (0.55)	0–4	404
Absolute difference per item^b^	0.10 (0.16)	0–1	404
SI^c^	0.13 (0.24)	0–1	404
SI tremor	0.06 (0.22)	0–1	404
SI bradykinesia	0.16 (0.29)	0–1	404
SI rigidity	0.07 (0.23)	0–1	400
Asymmetry (SI ≥ 0.20)	84 (20.8%)		404

*SI, symmetry index; UPDRS, Unified Parkinson Disease Rating Scale*.

*^a^The total of the items 20–26 of the UPDRS motor score is >0*.

*^b^The mean absolute difference between the left and right UPDRS motor items 20–26*.

*^c^Higher values of the symmetry index indicate a higher degree of asymmetry. Symmetry index of the UPDRS motor items 20–26 = (mean absolute difference UPDRS left and right)/(sum of the mean UPDRS left and right)*.

### Asymmetry in Parkinsonism in Relation to Severity of Psychopathology

The (multilevel) regression, controlled for predefined covariates, yielded both significant and non-significant coefficients between asymmetry in parkinsonism (symmetry index) and the severity of psychopathology (CGI-SCH SI subscales) with a range of 0.37–3.74 (patients *N* = 188, number of measurements *N* = 468), with a significant coefficient for the positive symptom scale (β = 3.74; 95% CI, 0.35–7.31, *p* = 0.031) (Table 4).

**Table 4:** Multilevel mixed model results: regression coefficients of asymmetry (symmetry index) with the severity of psychopathology (CGI-SCH SI) (patients *N* = 188, number of measurements *N* = 468)^a^.

	Symmetry index^b^			*N*

	β	95% CI	*p* value
**CGI-SCH SI**
Positive	3.74^c^	0.35 to 7.13	0.031	462
Negative	1.40	−2.07 to 4.88	0.429	462
Depressive	0.37	−2.24 to 2.98	0.780	465
Cognitive	1.07	−2.15 to 4.30	0.514	461
Global	2.31	−0.65 to 5.27	0.126	462

*CGI-SCH SI, Clinical Global Impression-Schizophrenia scale severity index; CI, confidence interval; UPDRS, Unified Parkinson Disease Rating Scale*.

*^a^Clinical assessments for all covariates in the model were completed in 468 measurements of 188 patients, with parkinsonism in the upper/lower limb(s) in at least one measurement*.

*^b^Higher values of the symmetry index indicate a higher degree of asymmetry. Symmetry index of the UPDRS motor items 20–26 = (mean absolute difference UPDRS left and right)/(sum of the mean UPDRS left and right)*.

*^c^Coefficient was considered significant (*p* < 0.05)*.

*The β coefficients in this table represent regression coefficients for the symmetry index as independent, and the CGI-SCH SI scales as dependent variables*.

*The β coefficients were corrected for the following covariates: age, sex, dosage of antipsychotics (defined daily dose), diagnosis (schizophrenia/other), concomitant anticholinergic use (yes/no), presence of movement disorders (parkinsonism, dyskinesia, dystonia, akathisia as continuous variables) and for the interaction between symmetry index and age*.

*The symmetry index was added as a squared covariate in the regression model, suggesting a non-linear relationship between the symmetry index and severity of psychopathology*.

## Discussion

Parkinsonism presented asymmetrically in 20.8% (84/404) of the measurements in our cohort of 190 long-stay psychiatric patients with chronic mental illness requiring long-term antipsychotic treatment. A new finding was an association between asymmetry in parkinsonism and the severity of psychopathology, which was positive for all subscales of the CGI-SCH SI and significantly associated with the positive symptom scale (β = 3.74, 95% CI = 0.35–7.31, *p* = 0.031).

The data of this study illustrate that DIP can present asymmetrically in a substantial part of patients although the disorder is classically described to appear symmetrically (5, 64). Therefore, asymmetry of parkinsonism does not provide diagnostic value for differentiating between DIP and iPD, in which asymmetric parkinsonism is classically described. These findings are consistent with previous reports of prevalence rates of asymmetry in DIP ranging from 18 to 54%, with most studies reporting rates of 30% or higher (21, 27, 44, 45, 64–67). The widely varying estimates can be attributed to the heterogeneity in study population, measurement scales and definitions of asymmetric parkinsonism. Furthermore, most studies did not use a validated measurement scale for parkinsonism, did not provide a case definition of asymmetric parkinsonism, and included a small number of patients, thus making it difficult to ensure a reliable estimate of the prevalence of asymmetric DIP.

Our second finding, of a relationship between asymmetry in parkinsonism and the severity of psychopathology in chronic psychiatric patients is, to the best of our knowledge, new and possibly clinical relevant. This finding is in line with the idea that brain asymmetry can be related to psychopathological manifestation of the disease. Namely, structural and functional findings of brain asymmetry have been linked to symptom severity in patients with schizophrenia, with positive (47–49) and negative (50–52) symptom severity, cognitive impairment (47, 49, 51), and duration of illness (48). These findings include increased and reduced asymmetry in several brain regions and both are related to increased severity of psychopathology (47–52). The findings of both increased and reduced brain asymmetry in schizophrenia may reflect the heterogeneity in the pathogenesis of schizophrenia or in the brain regions affected by reduced or increased asymmetry. In this study population of long-stay psychiatric patients, it could be hypothesized that increased brain asymmetry, contributing to the pathomechanism of the mental illness, could also result in asymmetry of parkinsonism. Nevertheless, as we did not perform neuroimaging, we can only speculate about the underlying pathomechanism of the relationship between asymmetry in parkinsonism and severity of psychopathology.

To our knowledge, this is the first study that explores the relationship between asymmetry in DIP and the severity of psychopathology. The occurrence of DIP by itself, as well as of other drug-induced movement disorders, has been related to psychopathology and cognitive functioning in patients with schizophrenia and other psychotic disorders (68–71). In adolescents at risk for psychotic disorders, movement abnormalities have been identified as an early risk indicator that may be predictive for conversion to psychosis and symptom severity (70, 71). The results of this study suggest that not only the presence but also asymmetry of DIP may be a risk indicator for the psychiatric patient for severity of psychopathology. Identifying clinical aspects of drug-induced movement disorders, such as asymmetry of symptoms, which can provide valuable information on the severity of illness, is of particular importance for clinical practice as assessment of movement disorders takes little time and can be easily implemented.

### Strengths

First, parkinsonism was (repeatedly) assessed on the UPDRS motor scale, a validated measurement tool with high test–retest reliability and internal consistency (72). The UPDRS motor scale provides severity ratings of tremor, bradykinesia, and rigidity of the left and right, upper and lower limbs separately, making this scale ideal for assessment of motor symptom asymmetry. Second, the assessments of all movement disorders were performed by a single person, a psychiatrist trained and retrained by an expert on movement disorders (senior author: PH), to prevent confounding of the results by reduced interrater or intrarater reliability. Third, we were able to obtain data from a cohort with a naturalistic and pragmatic design of a representative chronic psychiatric population (18, 73). Thus, the study yields results with a high external validity for clinical practice (73). Fourth, the importance of repeated measures, rather than a single cross-sectional measure, for movement disorders should be noted. Given the continuously fluctuating nature of movement disorders, a prospective study design may measure the phenotype more specifically. Therefore, this study design with repeated measures may result in a more stable phenotype, thus increasing the validity of the associations we found.

### Limitations

First, the approach used to quantify asymmetry in parkinsonism (the UPDRS motor symmetry index) may have led to biased results, as scores on the symmetry index tend to be low. Low index scores may be attributed to the limited range of the UPDRS motor scores (range, 0–4), which can easily produce ceiling effects. Furthermore, the symmetry index is a severity dependent scale, with relatively low scores when overall UPDRS motor scores are high. However, the symmetry index is considered as the most validated measurement for motor asymmetry and is preferred above other methods such as the absolute difference in side to side UPDRS motor scores (54, 55). In the current study population, overall UPDRS motor scores are relatively low (mean item score = 0.64, SD = 0.55), which makes the results unlikely to be biased by severity dependency or ceiling effects. Moreover, we were still able to identify a positive association between UPDRS asymmetry and psychopathology for all CGI-SCH SI subscales.

Second, the CGI-SCH SI is a brief assessment tool that may not be comprehensive or detailed enough to assess the severity of psychopathology properly. However, when compared to the Positive and Negative Symptom Severity (PANSS) scale, interrater reliability, sensitivity to change, and correlation coefficients between the instruments were high and comparable (57). Furthermore, the CGI-SCH SI might be more relevant for clinical practice, as it evaluates both symptom severity and interference with functioning, while the PANSS exclusively evaluates symptom severity (57).

Third, one may argue that Bonferroni correction for multiple testing should be applied. This would have resulted in a corrected critical *p* value of 0.01, with none of the results reaching significance. However, the Bonferroni correction assumes independency of the performed tests, which is probably not the case for this analysis as symptoms are mutually correlated. Furthermore, multiple testing corrections are designed to reduce type I errors (incorrect rejection of the null hypothesis) and thereby increase type II errors (incorrectly retaining of the null hypothesis) and greatly reduce power at the same time. Therefore, we chose not to correct for multiple testing but to accurately describe the performed tests and to discuss possible interpretations of the results instead (61–63). As this study concerned a hypothesis-driven research exploring a newly generated hypothesis, we considered the increase of type II errors undesirable. Furthermore, all beta coefficients for the CGI-SCH SI scales were estimated to be positive (range, 0.37–3.74), thus making it likely that asymmetry in parkinsonism is indeed positively related to psychopathology.

Finally, some authors may argue about the inclusion criteria for the regression analysis, as we did not use cut-off values for the case definition of parkinsonism. Instead, we included all patients presenting with parkinsonism in the lower and/or upper limb(s), even if symptoms were mild or were observed only once, to ensure a non-restrictive exploration of the association between asymmetry in parkinsonism and symptom severity. Noteworthy, *post hoc* analyses with more stringent inclusion criteria yielded nearly the same results. Therefore, the use of other inclusion criteria seemed irrelevant for the current analysis.

In conclusion, this study demonstrates a differential diagnostic and a new clinical relevant finding for psychiatric patients with DIP. Asymmetric presentation of DIP is quite common and therefore not useful to differentiate between DIP and other parkinsonian disorders. More important and clinical relevant is that asymmetry in DIP is significantly related to increased severity of psychopathology. If replicated, this could be of prognostic value to identify the more severely affected patient with possibly a poorer prognosis.

## Ethics Statement

This study was carried out in accordance with the recommendations of the “Medisch-ethische Toetsingscommissie Instellingen Geestelijke Gezondheidszorg” (Review Board for Human Research in Psychiatry) with written informed consent from all subjects. All subjects gave written informed consent in accordance with the Declaration of Helsinki. Consent obtained from the next of kin was neither necessary nor recommended by the Review Board for Human Research in Psychiatry. The protocol was approved by the standing Institutional Review Board, “Medisch-ethische Toetsingscommissie Instellingen Geestelijke Gezondheidszorg” (Review Board for Human Research in Psychiatry), the Netherlands (protocol number 377).

## Author Contributions

LP devised the main conceptual idea and discussed this with PB and PH. PB and PH encouraged LP to investigate asymmetry in parkinsonism and supervised the project. LP performed the statistical analysis with supervision of PB. LP wrote the manuscript with input from PB and PH. All authors discussed the results and interpretations of the results and contributed to the final manuscript. The authors agree to be accountable for all aspects of the work in ensuring that questions related to the accuracy or integrity of any part of the work are appropriately investigated and resolved.

## Conflict of Interest Statement

The authors declare that the research was conducted in the absence of any commercial or financial relationships that could be construed as a potential conflict of interest.
